# Capabilities of fecal calprotectin and blood biomarkers as surrogate endoscopic markers according to ulcerative colitis disease type

**DOI:** 10.3164/jcbn.18-92

**Published:** 2019-03-07

**Authors:** Hiroki Sonoyama, Kousaku Kawashima, Shunji Ishihara, Satoshi Kotani, Nobuhiko Fukuba, Akihiko Oka, Ryusaku Kusunoki, Yasumasa Tada, Yoshiyuki Mishima, Naoki Oshima, Ichiro Moriyama, Takafumi Yuki, Koji Onishi, Yoshikazu Kinoshita

**Affiliations:** 1 Department of Internal Medicine II, Shimane University Hospital, 89-1 Enya-cho, Izumo, Shimane 693-8501, Japan; 2 Inflammatory Bowel Disease Center, Shimane University Hospital, 89-1 Enya-cho, Izumo, Shimane 693-8501, Japan; 3 Cancer Center, Shimane University Hospital, 89-1 Enya-cho, Izumo, Shimane 693-8501, Japan; 4 Division of Internal Medicine, Matsue Seikyo General Hospital, 8-8-8 Nishitsuda, Matsue, Shimane 690-8522, Japan

**Keywords:** ulcerative colitis, fecal calprotectin, C-reactive protein, colonoscopy

## Abstract

Fecal calprotectin level in ulcerative colitis patients is correlated with endoscopic findings. However, its association with various ulcerative colitis disease types has not been elucidated. In the present study, we investigated the correlation of fecal calprotectin level with endoscopic findings as compared to blood biomarkers according to ulcerative colitis disease type. Fecal calprotectin as well as the blood biomarkers: C-reactive protein (CRP), white blood count (WBC), erythrocyte sedimentation rate (ESR), hemoglobin, platelet count (PLT), and serum albumin (Alb) were measured in patients who underwent a complete colonoscopy. Disease type was divided into proctitis, left-sided colitis, and extensive colitis. Correlations of fecal calprotectin and blood biomarker levels with Mayo endoscopic subscore were analyzed. A total of 186 colonoscopy examinations were performed in 124 patients with ulcerative colitis. Fecal calprotectin level showed a significant correlation with Mayo endoscopic subscore regardless of disease type (proctitis, r = 0.54, *p*<0.01; left-sided colitis, r = 0.75, *p*<0.01; extensive colitis, r = 0.78, *p*<0.01), and clearly discriminated inactive (Mayo endoscopic subscore 0) from active stages (Mayo endoscopic subscore 1–3). On the other hand, none of the examined blood biomarkers showed a correlation with Mayo endoscopic subscore in the proctitis group, while weak correlations of several biomarkers (CRP, WBC, ESR, PLT and Alb) with Mayo endoscopic subscore were found in left-sided colitis and extensive colitis cases. This is the first report to elucidate the capabilities of fecal calprotectin and blood biomarkers as endoscopic surrogate markers according to ulcerative colitis disease type.

## Introduction

Ulcerative colitis (UC) is an immune-mediated disorder characterized by mucosal inflammation in the colon and rectum, with affected patients demonstrating various clinical symptoms, including abdominal pain, diarrhea, melena, and fever.^(1–3)^ Although the intractability of this disease is widely recognized, novel therapeutic drugs such as biologics and tacrolimus recently developed have contributed to improvements in remission induction and patient health maintenance.^(4–8)^ Such newly established treatments are anticipated to lead to mucosal healing (MH) and induction of clinical remission, as well as reductions in the rates of hospitalization and surgical resection.^(9–11)^ As a result, evaluations of mucosal inflammation and healing have emerged as essential management procedures for clinical monitoring of UC patients.

A variety of methods are used for monitoring colonic mucosal inflammation associated with UC, with colonoscopy findings recognized as the gold standard. However, an endoscopic examination is relatively invasive and difficult to perform frequently in clinical practice. On the other hand, blood biomarkers, including C-reactive protein (CRP), white blood count (WBC), and erythrocyte sedimentation rate (ESR), are widely utilized as noninvasive biomarkers for the same purpose,^(12–14)^ though those findings are insufficient to precisely reflect endoscopic findings. In this regard, alternative noninvasive methods are considered necessary for evaluating endoscopic activity in UC patients.

Calprotectin, a complex of the proteins S100A8 and A100A9, represents most of the cytosolic proteins found in granulocytes, which are released into colonic lumina from inflamed mucosa during inflammation.^(15)^ Recent studies have shown that the level of fecal calprotectin (FC) is significantly correlated with disease severity as well as extent of affected mucosa in UC patients,^(16)^ and is considered to be superior to blood biomarkers such as CRP and WBC.^(17,18)^ Thus, measurement of FC level as a surrogate marker that reflects colonic inflammation associated with UC is recommended as a part of clinical examinations of patients.

In clinical practice, UC disease type is defined by the extent of affected mucosa and divided into 3 groups according to the Montreal classification;^(19)^ proctitis, left-sided colitis, and extensive colitis, which is helpful for assessment of disease activity as well as choice of treatment methods. On the other hand, the types of noninvasive markers that accurately reflect endoscopic findings according to disease type in UC patients has not been fully examined. It is considered that appropriate assessment of affected mucosa in terms of disease type may be useful for clinical management of patients with UC. In the present study, we enrolled UC patients and investigated the utility of FC level according to disease type as compared to conventional noninvasive blood biomarkers for predicting endoscopic findings.

## Methods

### Patients

This was a prospective study conducted from February 2013 to March 2017 at Shimane University Hospital and Matsue Seikyo General Hospital in Japan, and the protocol was approved by the institutional review board of both hospitals. Each patient provided written informed consent for participation. UC patients with a previously established diagnosis who underwent colonoscopy examinations were enrolled. Those who underwent a sigmoidoscopy, with a history of colorectal surgery or acute infectious enterocolitis, or with a regular intake of aspirin and/or other nonsteroidal anti-inflammatory drugs (NSAIDs) were excluded,^(20,21)^ as were those unable to provide a fecal sample. To reduce the differences between numbers of colonoscopy examinations for each patient, findings of up to 2 for each patient were included in the analysis. As for patient demographics, age, gender, disease duration, clinical activity, disease extent, and concomitant medications were noted at the time of the colonoscopy examination. Disease type was divided according to the Montreal classification into proctitis (E1), left-sided colitis (E2), and extensive colitis (E3), which was determined based on the greatest extent of affected mucosa in previous and present colonoscopy findings.^(19)^

### Clinical disease activity

Clinical disease activity was assessed on the day of the colonoscopy examination using total Mayo score, which is comprised of 4 sub-scores, including stool frequency, rectal bleeding, endoscopic findings, and physician’s global assessment, as previously reported.^(22)^

### Colonoscopy findings

Patients with UC received a polyethylene glycol- or magnesium citrate-based electrolyte solution for bowel preparation prior to the colonoscopy examination. All were performed by experienced colonoscopists using an EVIS 260 series device (Olympus, Tokyo, Japan). Colonoscopy findings were assessed according to the classification based on MES.^(22)^ Findings from an incomplete examination (cecum not reached) were excluded from analysis.

### Measurement of fecal calprotectin and blood examinations

Fecal samples were collected within the 3-day period prior to undergoing a colonoscopy and stored at −20°C until transfer to an external laboratory (SRL Inc., Tokyo, Japan) for analysis. FC level was determined using an enzyme-linked immunosorbent assay method (PhiCal^®^, Immundiagnostik AG, Germany). Blood samples were obtained on the day of the endoscopy prior to the procedure, with WBC, hemoglobin (Hb) level, platelet count (PLT), and ESR level, as well as serum high sensitive CRP and serum albumin (Alb) levels determined at the clinical laboratory of each hospital.

### Statistical analysis

Parametric numerical results are presented as the mean ± SD, while nonparametric values are presented as the median and interquartile range (IQR). Correlation analyses between MES and selected variables were performed with Spearman’s rank correlation test, with differences between those correlations analyzed using Fisher’s r to z transformation. A Mann-Whitney test and Kruskal-Wallis test were used to investigate differences between nonparametric data. All statistical analyses were performed using the SPSS statistical package (ver. 19.0, SPSS, Chicago, IL). All *p* values are two-sided, with *p*<0.05 considered to indicate statistical significance.

## Results

### Patients

A total of 186 complete colonoscopies accompanied by fecal sample collection were performed in 124 UC patients (79 males, 45 females). The baseline characteristics of eligible patients on the day of the endoscopy and colonoscopy findings are presented in Table 1. The extent of disease was determined according to the Montreal classification. Of the 186 colonoscopy examinations, 93 (50.0%) showed extensive colitis (E3), 54 (29.0%) left-sided colitis (E2), and 39 (21.0%) proctitis (E1). The numbers of patients treated with corticosteroids, tacrolimus, thiopurine, and biologics were greater in the E2 and E3 groups, while the number treated with topical aminosalicylate was higher in the E1 group. Other background factors were not different among the 3 groups.

### Correlations of FC and blood markers with MES according to ulcerative colitis disease type

The correlations of FC and other blood markers with MES according to UC disease type were statistically analyzed (Spearman’s rank correlation coefficient). As shown in Table 2, FC showed a significant correlation with MES regardless of disease type (E1, r = 0.54, *p*<0.01; E2, r = 0.75, *p*<0.01; E3, r = 0.78, *p*<0.01), though the correlation coefficient value was relatively low for the E1 group as compared to the E2 and E3 groups. Furthermore, significant correlations with MES were found for CRP (E3, r = 0.43, *p*<0.01; E2, r = 0.45, *p*<0.01), ESR (E3, r = 0.25, *p* = 0.02; E2, r = 0.32, *p* = 0.02), Alb (E3, r = −0.32, *p*<0.01), WBC (E3, r = 0.30, *p*<0.01), and Plt (E3, r = 0.33, *p*<0.01), though the correlation coefficient values were lower for each of those biomarkers as compared to that of FC. Only FC level had a significant correlation with MES in the E1 group. Moreover, we also investigated the correlation of partial Mayo score (stool frequency, rectal bleeding, physician’s global assessment) with MES and found a significant correlation regardless of disease type (E1, r = 0.74, *p*<0.01; E2, r = 0.68, *p*<0.01; E3, r = 0.73, *p*<0.01). In addition, FC showed a significant correlation with partial Mayo score in 3 disease types (E1, r = 0.62, *p*<0.01; E2, r = 0.50, *p*<0.01; E3, r = 0.71, *p*<0.01).

### Correlation of FC and CRP levels with MES in each disease type

**Fig. 1:**
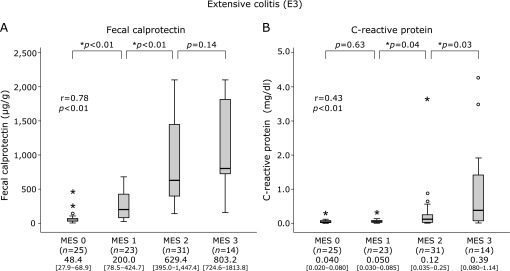
Boxplot showing median values and interquartile range for levels of fecal calprotectin (FC) and C-reactive protein (CRP) levels according to Mayo endoscopic score (MES 0–3) in patients with extensive ulcerative colitis (UC). A: FC. B: CRP.

**Fig. 2:**
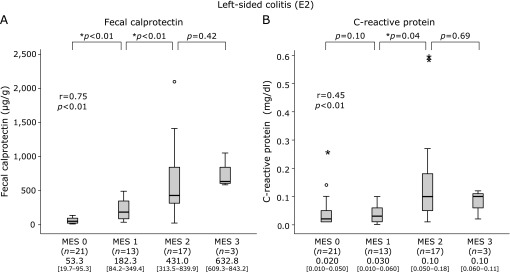
Boxplot showing median values and interquartile range for levels of fecal calprotectin (FC) and C-reactive protein (CRP) levels according to Mayo endoscopic score (MES 0–3) in patients with left-sided type of ulcerative colitis (UC). A: FC. B: CRP.

**Fig. 3:**
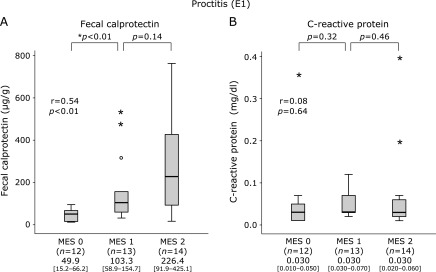
Boxplot showing median values and interquartile range for levels of fecal calprotectin (FC) and C-reactive protein (CRP) levels according to Mayo endoscopic score (MES 0–3) in patients with proctitis of ulcerative colitis (UC). A: FC. B: CRP.

As shown in Fig. 1, the median FC and CRP levels were elevated in association with MES in the E3 group, though no significant elevation of CRP was found in patients with MES 0 or 1. In addition, the median FC level was also elevated in association with MES in the E2 (Fig. 2) and E1 (Fig. 3) groups. Furthermore, median CRP was elevated in patients with MES 2 in the E2 group (Fig. 2), while no such elevation was seen regardless of MES in E1 (Fig. 3).

## Discussion

In recent years, management of UC has substantially changed in accordance with development of effective novel drugs. In particular, evaluation of the presence of mucosal inflammation and its healing has become recognized as an essential process for considering the necessity of therapeutic intervention,^(23,24)^ for which a variety of noninvasive blood and fecal biomarkers are used for assessment of mucosal activity in UC patients.^(25–27)^ Of those, FC has been found to be a reliable noninvasive marker that reflects endoscopic findings and its utility has been examined in comparisons with several different blood biomarkers.^(17–18)^ Schoepfer *et al.*^(17)^ investigated the correlations of FC, CRP, and WBC with Rachmilewitz endoscopic activity index, and found that FC level had the closest correlation with endoscopic disease activity, followed by CRP and WBC. Lobatón *et al.*^(28)^ also reported that FC was more closely correlated with MES than PLT, WBC, and CRP. Although those studies revealed the utility of FC as a surrogate marker of endoscopic activity, whether its level is associated with endoscopic activity according to UC disease type remained to be elucidated.

For the present study, we divided UC patients into 3 disease types based on the extent of affected mucosa and investigated the correlation of FC level with MES. Our results clearly showed that FC has a significant correlation with MES regardless of UC disease type. On the other hand, none of the blood biomarkers examined showed a correlation with MES in the E1 group, while weak correlations of several of those with MES were found in patients in the E2 or E3 group. Moreover, we examined the correlation of CRP with MES as compared to FC, which indicated the ability of FC to clearly discriminate inactive (MES 0) from the mildly active (MES 1) stage regardless of disease type, whereas CRP was not found useful for assessment of low-grade mucosal inflammation. This is the first report to show differential capabilities of FC as compared to conventional blood biomarkers according to UC disease type in a clinical setting.

Our results revealed that the examined blood biomarkers had a lower correlation with endoscopic findings in UC patients as compared to FC. However, the usefulness of blood biomarkers, such as CRP, ESR, Alb, WBC, and PLT, for monitoring of colonic inflammation in UC patients seems to be an important issue. An association with MES by those was only found in patients with E2 or E3 disease, but not in the E1 group, suggesting that extension of affected mucosa to a proximal site in UC is associated with increased CRP, ESR, PLT, and WBC, or a decreased level of Alb. In this regard, it may be possible to diagnose exacerbation of affected mucosa to a proximal site in clinical practice based on those blood biomarker changes. Recently, Osada *et al.*^(13)^ reported that CRP and ESR levels well reflected colonoscopy findings in proximal colon sites and recommended a colonoscopy examination for UC patients in clinical remission when those biomarkers are elevated. Our findings of the association of increased levels of CRP and ESR with the proximal extent of affected mucosa in UC support those results.

The present study demonstrated that FC has a significant correlation with colonoscopy findings regardless of UC disease type. However, the correlation coefficient value was relatively low for the E1 group as compared to E2 and E3. Since the total stool content of FC is reduced by the extent of narrowing of affected mucosa in proctitis cases, measurement accuracy might be lowered and make it more difficult to detect FC level differences among patients. In addition, the frequency of solid stool as compared to diarrhea is higher in proctitis as compared to the other disease types, which might lead to an uneven distribution of FC in stools and influence its measurement. In addition to biomarkers, we also evaluated using a partial Mayo score based on UC disease type and found a correlation with MES, even in patients with proctitis. Those findings suggest that assessment with that partial Mayo score in addition to FC level would be helpful for predicting endoscopic findings of proctitis in clinical practice.

Our study has several limitations. The examinations were conducted at 2 different hospitals in Japan. Although all colonoscopy findings were determined by experienced gastroenterologists, additional investigations at a greater number of centers will be necessary for reducing inter-observer variations. Second, in association with that first limitation, the number of patients with MES 3 in each group was relatively low in our cohort, which have might influenced the correlation analysis between biomarkers and MES. Third, it may be possible that the disease type is different with the extent of affected mucosa at the endoscopy. Because the disease type in each case was determined according to the most extensive area based on previous or present colonoscopy findings, however, it is considered unavoidable that the extent of endoscopic affected mucosa is not always the same as the disease type.

In summary, the present results revealed that FC level is significantly correlated with endoscopic findings regardless of UC disease type. On the other hand, none of the blood biomarkers examined showed correlations with endoscopic findings in UC patients with proctitis. We concluded that the utility of FC for predicting endoscopic activity in UC is superior to that blood biomarkers regardless of disease type.

## Figures and Tables

**Table 1:** Baseline characteristics of eligible patients on the day of the endoscopy according to extent of disease

	Extensive colitis	Left-sided colitis	Proctitis	*p* value
Total number of the endoscopy	93	54	39	
Age at the endoscopy (year), median, IQR	46.0 (33.0–60.0)	47.5 (30.0–61.0)	39.0 (32.0–63.5)	0.97
Duration of disease (year), median, IQR	5.3 (1.7–11.1)	7.6 (4.0–11.0)	7.0 (2.1–9.2)	0.78
Purpose of colonoscopy				
Evaluation of disease	49 (53%)	21 (39%)	23 (59%)	0.12
Surveillance	44 (47%)	33 (61%)	16 (41%)	
Clinical disease activity				
Remission stage	40 (43%)	31 (57%)	19 (48%)	0.24
Active stage	53 (57%)	23 (43%)	20 (52%)	
Concomitant medications				
Aminosalicylate	86 (92%)	51 (94%)	35 (90%)	0.70
Topical aminosalicylate	20 (22%)	12 (22%)	19 (49%)	<0.01
Corticosteroids	22 (24%)	4 (7%)	0 (0%)	N/A
Topical steroids	2 (2%)	2 (4%)	2 (5%)	0.66
Tacrolimus	12 (13%)	2 (4%)	0 (0%)	N/A
Thiopurine	41 (44%)	11 (20%)	0 (0%)	N/A
Biologics	15 (16%)	7 (13%)	0 (0%)	N/A
Colonoscopic findings				
Mayo endoscopic subscore 0	25 (27%)	21 (39%)	12 (31%)	0.10
Mayo endoscopic subscore 1	23 (25%)	13 (24%)	13 (33%)	
Mayo endoscopic subscore 2	31 (33%)	17 (31%)	14 (36%)	
Mayo endoscopic subscore 3	14 (15%)	3 (6%)	0 (0%)	

IQR, interquartile range; N/A, not applicable.

**Table 2:** Correlation of fecal and serological biomarkers with Mayo endoscopic subscore according to extent of disease

	Extensive colitis (*n* = 93)			Left-sided colitis (*n* = 54)			Proctitis (*n* = 39)	
	r	*p* value		r	*p* value		r	*p* value
Fecal calprotectin	0.78	<0.01		0.75	<0.01		0.54	<0.01
C-reactive protein	0.43	<0.01		0.45	<0.01		0.08	0.64
Erythrocyte sedimentation rate	0.25	0.02		0.32	0.02		−0.07	0.69
Albumin	−0.32	<0.01		−0.24	0.08		0.20	0.22
White blood count	0.30	<0.01		0.26	0.06		0.19	0.26
Hemoglobin	−0.12	0.25		0.10	0.49		0.17	0.31
Platelet count	0.33	<0.01		0.10	0.46		0.05	0.78

r, Spearman correlation coefficient.
